# Commissioning and co‐production in health and care services in the United Kingdom and Ireland: An exploratory literature review

**DOI:** 10.1111/hex.14053

**Published:** 2024-05-02

**Authors:** Rebecca J. Scott, Elspeth Mathie, Hannah J. H. Newman, Kathryn Almack, Louca‐Mai Brady

**Affiliations:** ^1^ Library and Computing Services University of Hertfordshire London UK; ^2^ Health and Social Care Inclusion, Centre for Research in Public Health and Community Care University of Hertfordshire London UK; ^3^ Applied Psychology, School of Life and Medical Sciences University of Hertfordshire London UK; ^4^ Family Lives and Care, Centre for Research in Public Health and Community Care University of Hertfordshire London UK; ^5^ Communities, Young People and Family Lives, CRIPPAC, Centre for Research in Public Health and Community Care University of Hertfordshire London UK

**Keywords:** commissioning, co‐production, health, health services, literature review, social care

## Abstract

**Introduction:**

This exploratory literature review seeks to examine the literature around commissioning processes in the co‐production of health and care services, focusing on two questions: How do health and care commissioning processes facilitate and/or pose barriers to co‐production in service design and delivery? What are the contextual factors that influence these processes?

**Method:**

A systematic search of three databases (Medline, Public Health and Social Policy and Practice) and a search platform (Web of Science) was conducted for the period 2008–2023. A total of 2675 records were retrieved. After deduplication, 1925 were screened at title and abstract level. Forty‐seven reports from 42 United Kingdom and Ireland studies were included in the review. A thematic synthesis of included studies was conducted in relation to the research questions.

**Results:**

The review identified one overarching theme across the synthesised literature: the complexity of the commissioning landscape. Three interconnected subthemes illuminate the contextual factors that influence this landscape: commissioners as leaders of co‐production; navigating relationships and the collective voice.

**Conclusion:**

Commissioning processes were commonly a barrier to the co‐production of health and care services. Though co‐production was an aspiration for many commissioners, the political and economic environment and service pressures meant that it was often not fully realised. More flexible funding models, longer‐term pilot projects, an increased emphasis in social value across the health and care system and building capacity for strong leadership in commissioning is needed.

**Patient and Public Contribution:**

Patients and the public did not contribute to this review as it was a small piece of work following on from a completed project, with no budget for public involvement.

## 1 INTRODUCTION

Co‐production is now a mandatory expectation of delivering health and care services^1^ become a commonly used ‘buzzword’.^2^, ^3^ Co‐production of public service delivery relates to the equal participation of service users, community representatives and professionals in the design and delivery of services.^4^ There are many definitions of co‐production^5^; it can be seen as both an approach to service delivery and an underlying set of values and principles. The Co‐Productive Collective adopt approaches that illustrate the core values underpinning co‐production; these are about being inclusive, transparent, kind, reflective and embracing change.^6^ Other essential values include power sharing, mutual respect as well as addressing diversity.^7^ An inclusive approach to co‐production centres upon removing barriers so that all members can participate fully in the process of service design and delivery.^8^ Prioritising diversity ensures that different perspectives, experiences, and ways of knowing are recognised and valued.^9^ Our commitment to honest and critical reflection is nurtured through transparent communication between health and care professionals and service users, fostering meaningful collaboration at every stage of the co‐production cycle.^7^

Drawing on the authors' previous work, this review defines co‐production as: ‘an approach in which practitioners and the public/communities/service users work together in equal partnership, sharing power and responsibility from the start to the end of a project, including planning, delivery, and evaluation’.^10^ In reality, co‐production occurs at different stages of the service design cycle.^11^ It is acknowledged that the ‘ideal’ of co‐production, such as working in equal partnership throughout, is difficult to achieve and measure.^12^

Co‐production is often seen as the ‘gold standard of public involvement, as opposed to more consultative approaches^13^; however, some have expressed concern that the increasing interest in co‐production comes at the expense of service user and community‐led approaches,^14^, ^15^ It remains to be seen whether services can be routinely delivered in a truly co‐productive manner given the context and structures in which they operate.^9^, ^16^, ^17^

Co‐production is instrumental in moving towards a place‐based approach to health and well‐being which requires a transfer of power from health and care bodies to people and communities and a focus on outcomes not outputs.^18^ The core principles of place‐based approaches align with co‐production values; namely, an emphasis on people, on celebrating and making the best use of local assets and a focus on prevention of ill health.^18^, ^19^

The term ‘co‐production’ is frequently used in service delivery and health and social care research, however, it has been less explored in terms of commissioning services and what facilitators and barriers there are. If service delivery is to move towards becoming more ‘co‐productive’, it is vital to examine the commissioning process.

### 1.1 Commissioning

In the United Kingdom, governance, management and funding of health and care systems are decentralised to England, Wales, Scotland and Northern Ireland. NHS England defines commissioning as ‘the process of assessing needs, planning and prioritising, purchasing and monitoring health services, to get the best health outcomes’.^20^ Health and care commissioning in England has undergone significant organisational and legislative changes over the last three decades due to periodic transitions in political leadership.^21^ The NHS Long Term Plan^22^ set out plans for integrated care systems (ICS) across England from April 2021. The 2022 Health and Care Act introduced new and widespread reforms; Clinical Commissioning Groups (CCGs) were replaced with ICS.^23^ Forty‐two integrated care boards now have statutory responsibility for commissioning services within their respective ICS and have a substantial share of the budget.^23^, ^24^ ICS also assumed responsibility from NHS England for commissioning primary care services.^24^ Co‐production is key to the delivery of ICS strategy.^25^ ICSs are based on the idea that collaboration between the NHS, social care, voluntary, community or social enterprise organisation (VCSE) organisations and communities is needed to improve local services and make the best use of public money.^26^ The aim is to ‘build positive and enduring partnerships with people and communities to improve services and outcomes, including engagement, co‐design and co‐production’.^27^

In Wales, there are seven Local Health Boards with commissioning and operational responsibility for health services; primary care is delivered through contracting with GPs, pharmacists, opticians and dentists.^28^, ^29^ Social care in Wales (as in England and Scotland) is funded through grants to local authorities and council tax revenue.^30^, ^31^, ^32^ The co‐production of health and care services is a core principle in the Social Services and Well‐being (Wales) Act 2014.^33^

In Scotland, health policy has remained largely stable.^31^ Fourteen regional NHS Boards are allocated funding to plan and provide healthcare for their local population.^34^ The Scottish Government introduced legislation underpinned by co‐production to integrate health and social care in 2016. However, inconsistent approaches to the implementation of co‐production of services are evident.^35^ Proposed new legislation would see a pivotal transfer of funding from local authorities to a National Care Service (NCS) using a central model of Care Boards for commissioning.^36^ Transitioning away from localised funding for social care has the potential to provide greater consistency of service provision across Scotland^36^; however, a less localised approach could fail to meet the needs of each distinct locality. NCS is adopting a co‐design approach to its development and a key policy strand is the Design School which will focus on building capacity for co‐production.^37^ If successful, future social care funding for Scotland will be built on a strong foundation of co‐production approaches.

Northern Ireland established fully integrated health and social care in the 1970s.^31^ Political challenges around the power‐sharing agreement have resulted in minimal policy changes.^31^ One Health and Social Care Board is responsible for commissioning with services delivered by five health and social care trusts.^31^ Co‐production of health and care services has been a key policy promise since 2016 in Northern Ireland.^38^

Across the UK, co‐production is recognised as a core principle in health and care policy and key to the integration of health and care. However, the political environment, legislative changes and pressures on services result in significant challenges for commissioners.^35^ The operationalisation of co‐production may be affected by how these different funding streams are organised and their associated requirements.

The third sector/VCSE also feature in the UK health and care market. Funding streams for these providers are local authority grants and/or contracts as well as health and community‐focused grant funding organisations.^39^, ^40^ For the purpose of this review, publicly funded health and care services in all settings (e.g., primary and secondary care, mental health, public health) and VCSE interventions are of interest.

### 1.2 Background

The rationale for this review was underpinned by the findings of an embedded ethnographic evaluation study of a third sector organisation delivering weight management and lifestyle change programmes^10^ which explored the potential for the co‐production of services. Key findings from this study identified commissioning processes, including short‐term funding cycles, uncertain funding and outcome‐based key performance indicators, as a fundamental barrier to the co‐production of health and care services.^10^ The findings also suggested that a cultural shift in public health commissioning, including longer funding cycles, more secure funding, and investment in people and communities could be a potential facilitator to co‐production. However, further exploration was needed to assess the extent to which this had also been found in the wider health and care context. This exploratory literature review therefore sought to ascertain if other studies in the co‐production academic and grey literature had identified similar findings.

### 1.3 Aims

The aims of this review were to explore the literature around health and care commissioning processes and how these can support or create barriers to co‐production. The review sought to answer the following questions:

### 1.4 Research questions

1. How do health and care commissioning processes facilitate and/or pose barriers to co‐production in service design and delivery?
2. What are the contextual factors that influence these processes?

The review will adopt a thematic synthesis to examine the literature in relation to the research questions.

## 2 METHODS

### 2.1 Search strategy

Searching for a systematic review in co‐production and commissioning is considered challenging due to the ‘disparate and scarce nature of evidence’ in this area.^41^ The multitude of definitions of co‐production,^5^ the need for bespoke approaches in co‐production^42^ and what is considered ‘misappropriation’ of the term^9^ add to the complexity of literature searching for this topic. Recognising the potential pitfalls, the present exploratory review employed a search in four databases and utilised additional methods including academic search engines (Google Scholar, BASE), manual and automated citation searching,^43^ and a literature mapping tool.^44^, ^45^

Three concept groups were used to structure the search:

1. Co‐production in health or social care services.
2. Commissioning.
3. Barriers/enablers.

Search terms for the co‐production of health and care services were developed from an existing scoping review of definitions^5^ and a search filter for patient and public involvement.^46^ Commissioning terms were developed through a scoping search and cited searching with an initial set of papers in an online tool.^45^ There is not an established medical subject heading for commissioning of healthcare services. Thus, related thesauri terms were identified using an online tool for analysing subject headings.^47^ A published search filter for countries similar to the United Kingdom was utilised to reduce the number of records for screening.^48^

An increasing trend of applied and conceptual research around co‐production guided the selected 2008–2023 date range for the search.^5^ The following databases were utilised:

1. Medline via Ovid (*n* = 834).
2. Social Policy and Practice via Ovid (*n* = 676).
3. Public Health via Proquest (*n* = 245).
4. Web of Science (*n* = 920) (see Supporting Information S1: File 1 for indexes).

Searches were run between 16 and 17 March 2023. A total of 2675 records were retrieved from database searches. After deduplication, 1925 records remained. Subsequently, records were uploaded into software for screening.^49^ Searches are presented in Supporting Information S1: File 1.

#### 2.1.1 Inclusion criteria

2.1.1.1

1. Focused on health and care services developed with, or by, people or communities.
2. Addressed the commissioning of services.
3. Publications from the United Kingdom and Ireland.

At the outset of the review, the research team planned to include publications from countries with healthcare contexts similar to the United Kingdom. However, it became evident during screening that our search retrieved predominantly publications from the United Kingdom and Ireland; therefore, we narrowed the focus.

The research team judged that consultation or engagement approaches considered lower on the ladder of citizen participation,^13^, ^50^ which identified barriers or enablers in commissioning, would also have relevance for the co‐production of services.

#### 2.1.2 Exclusion criteria

1. How‐to guides.
2. Brief reports where a full report was available.
3. Focused on broader public services (e.g. housing).
4. Articles focusing on commissioning of health and care *research*.
5. Priority setting methods.

### 2.2 Screening

**Figure 1:**
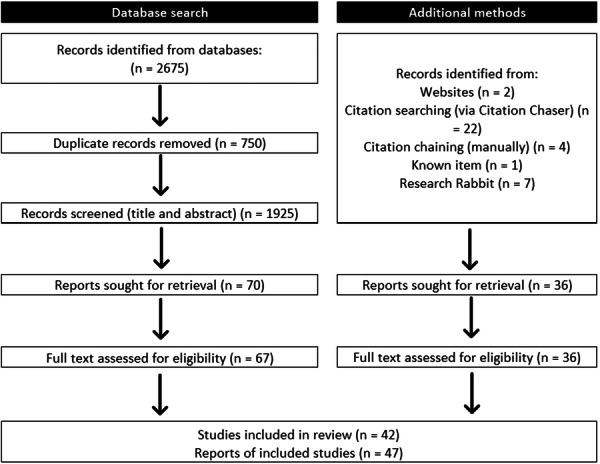
Exploratory search approach adapted from Preferred Reporting Items for Systematic Reviews and Meta‐analyses flow diagram.^51^, ^52^

Title and abstract screening in online software^49^ focused on health and care services developed with, or by, people or communities *and* commissioning. R. J. S. screened all publications at title/abstract level. H. J. H. N. screened 10% to check for accuracy. See Figure 1 for details of the study selection process.

### 2.3 Thematic synthesis

This review used Thomas and Harden's approach to qualitative thematic synthesis^53^ as it sought to reach new insights by synthesising and analysing primary studies together as a body of knowledge.^54^ The first stage involved inductive line‐by‐line coding in Nvivo. All text labelled as ‘findings’, ‘results’ or ‘recommendations’, in the body of the publication, were coded in relation to health and care commissioning processes.^53^ Next, codes were organised into a hierarchical structure, focusing on commonalities to shape descriptive themes (e.g., leadership, funding and flexibility). Finally, analytic themes were developed in relation to the research questions, that is, barriers and/or enablers of co‐production.

## 3 RESULTS

The review identified 47 publications (journal articles *n* = 23; grey literature reports *n* = 23; PhD thesis *n* = 1) from 42 studies. Publications originated from England *n* = 32,^55^, ^56^, ^57^, ^58^, ^59^, ^60^, ^61^, ^62^, ^63^, ^64^, ^65^, ^66^, ^67^, ^68^, ^69^, ^70^, ^71^, ^72^, ^73^, ^74^, ^75^, ^76^, ^77^, ^78^, ^79^, ^80^, ^81^, ^82^, ^83^, ^84^, ^85^, ^86^ United Kingdom *n* = 10,^87^, ^88^, ^89^, ^90^, ^91^, ^92^, ^93^, ^94^, ^95^, ^96^ Scotland *n* = 2,^97^, ^98^ Ireland *n* = 2^99^, ^100^ and Wales *n* = 1.^101^ Publications were largely qualitative (*n* = 40) or mixed methods (*n* = 4).

Eighteen publications describe their approach to working with service users or communities as co‐production and three as co‐design. See Supporting Information S2: Appendix 1 for an overview of included studies. Table 1 broadly summarises the types of services in the included studies.

**Table 1:** Broad overview of services in the included studies.

Type of service	Number of reports
Mental health	11
Commissioning	9
Older adults	6
Disabilities	3
Public services	3
Addiction services	2
Primary care	2
Social care	2
Arts for health	1
Cancer care	1
Healthcare systems	1
Public health	1
Long‐term conditions	1
Respiratory	1
Rural health	1
Transplant	1
Veteran services	1
Total number	47

In relation to the first research question regarding how health and care commissioning processes facilitate and/or pose barriers to co‐production, the review identified one overarching theme which spanned the synthesised literature: the complexity of the commissioning landscape. The second research question, exploring the contextual factors shaping commissioning processes, is addressed through three interconnected subthemes: commissioners as leaders of co‐production, the navigating relationships and the collective voice.

### 3.1 Complexity of the commissioning landscape

This overarching theme explores the inherent complexity in the commissioning landscape due to funding cycles and models, time, governance processes, outcomes monitoring and decommissioning and how these can constrain co‐production.

Funding cycles and models were a consistent barrier to co‐production with tendering processes considered complex, timescales unfeasible and restrictions on what is eligible for funding a challenge.^89^, ^91^, ^93^ Funders often required detailed planning for service implementation which limited the scope for/of co‐production.^67^, ^89^, ^93^ Insecure funding presented a significant risk to organisations and was a source of instability.^68^, ^71^, ^72^, ^78^, ^81^, ^91^ The rapidly changing sociopolitical environment meant commissioners themselves operated in a climate of uncertainty and experienced unexpected cuts in funding.^78^, ^89^ The sustainability of co‐produced services was considered essential, and organisations recognised that adequate funding was needed on an ongoing basis to support community‐led initiatives.^79^, ^85^, ^100^ However, the literature did not provide details around the minimum funding levels required for co‐produced projects. Often funders expected community‐led activities to move to business as usual. Though this can be the case,^74^, ^79^ there are examples of services that cease to exist.^69^, ^99^

Further complexity resulted from rigid commissioning structures. Lack of integration between health and care budgets, not enough focus on prevention, early intervention or whole system approaches meant limited funding was directed towards services co‐produced with communities.^69^, ^72^, ^79^, ^84^, ^86^, ^90^, ^94^, ^96^ The emphasis on short‐term goals and funding of pilot projects, which were popular with commissioners, hindered co‐production approaches.^64^, ^72^, ^79^, ^93^

Co‐production was understood to be time and resource intensive.^57^, ^62^, ^63^, ^66^, ^68^, ^76^, ^79^, ^82^, ^84^, ^89^, ^99^ The funding of longer‐term pilot projects was considered an enabler.^57^, ^64^, ^65^, ^74^, ^79^ This facilitated sufficient time to recruit participants from different parts of the community (including seldom heard groups), acclimatise to new ways of working and develop the service at a community‐driven pace.^64^, ^67^, ^69^, ^76^, ^79^, ^96^, ^101^ Flexibility in funding cycles and models was also a key factor in successful co‐production. Suggestions for improving funding flexibility in the literature included:

1. Less onerous structures, frameworks and application processes.^57^, ^89^
2. Aligning payment of providers with their operational needs (e.g., paying quarterly rather than annually).^96^
3. Using grant funding mechanisms to support smaller organisations with less experience of contracts and associated rigorous monitoring requirements.^89^
4. Providers of different services (e.g., social care, NHS, VCSEs) sharing resources and having freedom to pool budgets.^94^
5. Alliance and partnership approaches to deliver services^76^, ^82^, ^89^; For example, Lambeth's Living Well Network, an alliance contract between the third sector, a mental health trust, the CCG and the local authority.^76^

Despite a recognised need for flexibility, governance and financial accountability of co‐produced services and monitoring outcomes were deemed necessary.^59^, ^80^, ^89^, ^95^ Governance structures were sometimes viewed as ineffectual, an exercise in bureaucracy and in conflict with the values of co‐production.^60^, ^80^, ^81^ Community representation in contract monitoring is important for assessing compliance from a user perspective.^80^, ^95^ However, some community members found committee documents inaccessible and lengthy.^61^, ^80^, ^88^ The outcomes of co‐produced services were considered difficult to measure.^66^, ^69^, ^79^, ^81^, ^84^, ^91^ Preventative services and early intervention can lead to cost savings elsewhere in the health and care system but there was no simple method of demonstrating this.^84^, ^93^

Outcomes‐based commissioning is a whole systems approach to the commissioning, design and delivery and evaluation of services, a central feature is the focus on the health and wellbeing outcomes important to the local population rather than individual interventions.^102^ Community members, organisations, professionals and commissioners all agreed that evaluation was essential.^63^, ^70^, ^81^, ^95^ The transition to outcomes‐based commissioning was seen as a key enabler with successes measured in terms of the impact on individuals and communities.^89^, ^97^ It is suggested that the social return on investment is given increased weighting in commissioning rather than focusing solely on cost.^65^, ^67^, ^69^, ^94^, ^97^ Training and shared learning are potential facilitators of an effective shift to a model of outcomes‐based commissioning.^65^, ^67^ However, at present, outcomes‐based commissioning was largely an aspiration rather than established practice.^89^

Decommissioning and disinvestment are a fundamental part of the commissioning landscape.^61^, ^76^ Services deemed to no longer meet the evolving needs of the population are closed.^73^, ^100^ This can lead to new opportunities; for example, a commissioning decision to close a day centre led to the development of a peer support model to better support mental health patients' recovery. ^69^, ^97^ Commissioners are more likely to engage with co‐production where it involves reshaping existing services.^65^, ^90^ However, decommissioning is often unpopular and potentially harmful to some communities and to the principles of co‐production, such as building long‐lasting relationships.^58^, ^69^, ^70^, ^76^, ^77^, ^96^

The size and scale of providers were also shaped by the changing landscape of commissioning. Some commissioners preferred to work with larger organisations as it reduced the number of contracts and potentially simplified a care pathway.^89^ Whereas others felt the reduction in choice of providers reduced quality.^84^ Small bespoke approaches are synonymous with co‐production where services are designed with communities and local assets.^80^ This creates challenges for scaling up as the approaches are inherently place‐based and not necessarily transferable. It was difficult for smaller providers to build capacity for co‐production in their workforce.^89^, ^91^, ^98^ One proposed solution is alliance commissioning where a larger provider enters a partnership with several smaller providers. This was found to work well when partnerships start small and grow over time but required clear governance structures.^76^

### 3.2 Commissioners as leaders of co‐production

The first subtheme focuses on the role of commissioners in driving forward the co‐production of health and care services. It examines how their leadership influences the commissioning landscape as well as the personal values that foster this approach.

Commissioners with a clear understanding of co‐production principles demonstrated leadership creating a climate for co‐production to take place.^67^ These leaders excel at facilitating a shared understanding of population‐level health as well as local assets.^61^, ^66^, ^84^, ^95^ Leadership manifested in a longer‐term vision for co‐produced services which allowed these approaches to flourish over time.^64^, ^66^ Supportive commissioners were committed to building local capacity to ensure transformational change.^89^, ^96^ They were willing to listen, take risks, acknowledge failures and were open to doing things differently.^57^, ^73^, ^88^, ^101^ It was recognised that future commissioners who value co‐production approaches will need to be nurtured.^66^, ^82^

However, the sociopolitical climate in which commissioners operated was a significant challenge to developing this transformational leadership.^62^ Commissioners were subject to centrally driven mandates.^60^, ^62^ Pressures on the health and care system took precedence over co‐production when budgets are stretched.^65^, ^66^, ^68^, ^69^, ^73^ For many commissioners there was a tension between what was feasible and affordable and the perceived expectations of community members co‐designing services.^61^, ^66^, ^68^, ^90^, ^101^ Further, retention of experienced, supportive commissioners was a concern as changes in leadership could derail co‐production of services.^63^, ^68^

### 3.3 Navigating relationships

The second subtheme explores the importance of relationships within the commissioning landscape. It examines communication, power dynamics and trust between different stakeholders involved in the co‐production of services.

Nurturing relationships between stakeholders cultivated co‐production.^84^ This involved open dialogue and embracing the more challenging elements of the co‐production process (such as conflict and previous failures).^73^, ^74^, ^76^ Listening and effective communication were key to developing mutual understanding between commissioners, providers and communities.^61^, ^66^, ^74^, ^77^, ^89^, ^99^ This alleviated some concerns around unrealistic expectations due to financial constraints.^42^ Mature relationships which developed over an extended period were considered best placed to build co‐production approaches.^65^, ^66^, ^82^, ^89^ Collaborations with new partners were welcome; however, navigating new relationships in the complex landscape of commissioning was difficult for smaller, less experienced providers.^64^, ^89^, ^91^

Clarity of purpose and shared vision of the proposed health and care outcomes to be achieved and the scope and capacity to deliver them were central to effective co‐production.^59^, ^61^, ^62^, ^88^ Where this was lacking, it fostered distrust.^58^, ^61^, ^69^, ^88^, ^98^ Service users and communities valued transparency and regular feedback to understand how their involvement impacted the design and delivery of services.^58^, ^59^, ^60^, ^61^, ^65^, ^71^, ^77^, ^98^ Accessible and more informal communication methods were considered helpful to make meaningful contributions to decision‐making. Positive relationships afforded opportunities to influence others,^63^, ^75^, ^77^, ^80^ but this was sometimes tempered by rigid organisational structures and financial constraints.^61^, ^62^

A rebalancing of power is central to co‐production. This required a change in commissioners and providers' perspectives to recognise the strengths and assets of service users and communities.^64^, ^82^, ^95^ A successful shift of power to service users and communities positioned them as co‐producers and co‐designers^67^ and critical partners.^68^ Empowerment was evident in service users feeling heard,^83^ active decision‐making^93^ and a more strategic approach to their involvement activity.^71^ Examples of service users and community leaders independently pursuing their own ideas suggest a transfer of power can occur.^68^, ^77^, ^79^ Some commissioners and providers felt service user presence made people in powerful positions more willing to listen to ideas,^57^ and their voices added legitimacy to a shift in how services are designed.^61^, ^86^ Yet navigating the shift in power dynamics was not straightforward. Disempowerment of service users manifested in multiple ways: their apparent low status in meetings,^59^, ^60^ how meetings were conducted,^58^ limited opportunity to contribute their voices,^71^ lack of budget for their involvement activity,^61^ and a devaluing of their lived experience.^59^ Negative, discriminatory attitudes were evident as some staff, organisations and commissioners resisted the shift in power to service users.^56^, ^68^, ^80^, ^88^

Building mutual trust in relationships was vital for moving towards co‐production approaches. It was recognised that this required time and commitment from all stakeholders.^62^, ^66^, ^68^, ^88^ Community leaders were instrumental in building trust within their communities.^74^ Trust was fragile and vulnerable to changes in staffing and leadership.^60^ Previous tokenistic experiences of involvement and engagement approaches resulted in mistrust.^70^, ^71^ Some service users questioned whether their involvement was valued,^60^, ^71^ if it had any influence on commissioners' decision‐making,^63^ and in some cases believed it was used to legitimise unpopular decisions.^77^

### 3.4 The collective voice

This third subtheme centres around the collective voice of service users and communities in the co‐production of health and care services. It explores the issues of representation and inclusion, reward and recognition as well as the burden of involvement.

Lack of representation (or diversity) from a broad cross section of the population was a repeated concern in the literature.^56^, ^62^, ^66^, ^68^, ^73^, ^80^, ^100^ It was perceived that the service users most involved in co‐production and commissioning activities were a homogenous group consisting of white, middle class, retired individuals.^62^, ^68^, ^80^ Increasing diversity was considered critical to ensure services meet the needs of seldom heard and marginalised groups and address health inequalities.^66^, ^70^ It was also deemed important that service user representation in commissioning reflected the evolving demographic profile of the local area.^61^ Service users recognised that their role was to act as a conduit for the collective voice and represent the many different viewpoints within their communities.^77^, ^100^ As there is no template for an average service user, the commitment and lived experience insights of those who contribute should be embraced,^87^ while simultaneously developing active approaches to hear seldom‐heard voices. Stigma and bias hindered inclusion; for example, service users could be considered too enthusiastic or opinionated.^68^ One study identified a bias against disabled people which suggested that they would focus solely on their own concerns.^88^ Another reported how one service user was not permitted to raise issues of race.^71^

Service users' skill level was identified as important for successful involvement in commissioning.^68^ Training service users to develop knowledge of commissioning processes and codesign approaches was considered a key mechanism to drive change.^61^, ^63^, ^67^, ^69^, ^73^, ^77^, ^82^, ^87^, ^88^, ^100^ Some studies caution against professionalisation of service users but argue instead for recognition of the lived experience contribution.^87^, ^93^ Service users wanted to develop this knowledge as it was informative,^77^ symbolic of the value of their contribution^73^ and prepared them for expressing their views and experiences.^63^

Financial reward of service users and community members' contributions was a sensitive issue.^61^, ^73^, ^80^ Though professionals and commissioners were largely in agreement that it was important to pay service users and community members for their time,^77^, ^80^ it was not straightforward.^73^ Some service users were content to have their expenses met.^61^, ^63^ Some felt payment demonstrated the value of their contributions.^77^ Others felt payment would incentivise people to contribute for less altruistic reasons.^61^ Without payment for their time, a cycle of excluding those who do not have the resources is perpetuated.^71^, ^90^

The literature identified the burden of commitment that service users and community members can experience in the co‐production of health and care services. Emotional and physical fatigue,^63^, ^73^, ^75^, ^77^, ^87^ and the weight of responsibility were acknowledged.^80^ When involvement approaches were tokenistic, it was harmful to both individuals and communities.^61^, ^71^, ^77^, ^101^

## 4 DISCUSSION

This discussion synthesises the review findings with the wider literature on commissioning and co‐production. Initially, it explores the complexity of the commissioning landscape theme within the broader context of health and care policy. Next, it considers the role of commissioners as leaders of co‐production in a changing sociopolitical climate. Lastly, it outlines the changes needed to commission processes in relation to the navigating relationships and collective voice.

This exploratory review found that the complexity of the commissioning landscape constrains co‐production in health and care service design and delivery. Funding cycles and models, unfeasible timescales, rigid commissioning and governance structures are consistently reported as significant barriers. The review found examples of these constraints across the United Kingdom and Ireland.^89^, ^91^, ^93^, ^97^, ^98^, ^100^ The literature demonstrates that though different mechanisms of health and care commissioning exist, the challenges related to commissioning processes and co‐production are widespread and commonplace. The inclusion of involvement and engagement approaches in the review demonstrates that these challenges exist throughout all levels of the citizen participation ladder.^50^ There is a gap between the rhetoric of policy makers who mandate co‐production and place‐based partnerships, intended to meet the needs of local populations, and a rigid commissioning system which has not yet evolved to make it a reality.

The commissioners who act as ‘leaders’ of co‐production subtheme demonstrated that commissioners can create a climate for co‐production to thrive.^61^, ^66^ A willingness to radically rethink how services are designed is synonymous with commissioners who are supportive of co‐production. However, in their recent study of leaders in health and care integration in Scotland, Connolly et al. found that complexity in governance processes and the accelerated pace of health and social care service integration were barriers to co‐production.^35^ Though commissioners may wholeheartedly agree with the principles of co‐production, the wider sociopolitical climate constrains their capacity to translate them into action. Large‐scale transformations of health and care systems may require additional time to ‘bed in’ and move from being a barrier to an enabler of co‐production.

As outlined in the introduction, commissioning is vulnerable to transitions in political leadership and considerable financial constraint,^35^ operating in an environment of uncertain and changing economic and social conditions. To move co‐production from an aspiration of supportive leaders who ‘get it’ to a fundamental and embedded pillar of commissioning practice, substantial structural reform is needed. This includes:

1. increasing the time for the initial phase of co‐production projects,
2. allowing flexibility in implementation plans,
3. funding projects and organisations on a longer‐term basis,
4. building capacity for co‐production in the health and care workforce, as well as in voluntary and community organisations and
5. minimising restrictions on what and who is eligible for funding.

The provision of funding for the ongoing costs associated with co‐produced projects was considered important for sustainability; however, it is unclear what a minimum level of funding would look like in practice. There is a pressing need for commissioners to be afforded sufficient time and opportunities to develop effective strategies for facilitating its implementation.

The navigating relationships subtheme highlighted the importance of trust, communication and a rebalancing of power in effective co‐production. Commissioning processes can support a more equal distribution of power by ensuring operational costs are met; even small‐scale projects have costs associated (e.g., hall hire, equipment hire, staffing, transport, facilitation). Social value needs to be given greater emphasis in commissioning decisions so that preventative and early intervention approaches receive adequate funding. This is ever more pressing as the projected increase in anxiety and depression, diabetes and chronic pain are largely treated in the community and preventative approaches can improve quality of life.^103^ The voices of community members must be central to health and care commissioning if the system is to meet the projected rise in demand.

The collective voice subtheme identified that reward and recognition of service users' contributions were necessary for ensuring inclusivity in co‐production. A clear, transparent, properly costed and funded approach to costing co‐production will improve the inclusion of diverse people and communities in commissioning and underline the deeply valuable contribution members of the public make, as well as reduce the risk of tokenistic approaches. Rewards and recognition for members should be underpinned by clear payment guidelines for time and contribution,^71^, ^104^ such as those of the National Institute for Health and Care Research (NIHR) and the UCL Co‐production Collective.^105^, ^106^ A range of options for payment should be factored into these guidelines to accommodate differing circumstances of contributors.^107^ Similarly, consideration should be given to costing in time and expertise which may be needed to support public and community involvement and co‐production in commissioning.

The term ‘co‐production’ is now being written into strategic planning of health and care policies and services in many countries^25^ including the four UK nations and is generally viewed as a positive way to work with communities to improve health and social care services.^41^, ^108^, ^109^, ^110^ While the enablers and barriers to co‐production in research have been well documented, co‐production at the system level of commissioning must now be prioritised if services are to address health inequalities and meet the needs of the people. Future research could compare co‐production and system level commissioning in different international contexts.

### 4.1 Limitations

We were unable to conduct a full systematic review due to limited resources. The review findings are limited to a small sample of publications from the United Kingdom and Ireland. All were English language publications. The results are likely to have been influenced by database selection, the complexities associated with the concept of co‐production, and the specific terms chosen.

## 5 CONCLUSION

Policy makers recognise that co‐production of health and care services is central to aligning services to meet the population's needs. However, this rhetoric is more an aspiration than an operational reality due to sociopolitical and economic factors. This review has demonstrated that the current model of commissioning and its associated funding cycles and governance structures are commonly in conflict with a co‐productive approach. This disconnect between rhetoric and reality indicates a need for significant reform within the commissioning system to effectively address the health and care needs of the population.

The projected increase in demand on health and care services requires a swift transition to an outcomes‐based commissioning model. To deliver this transition, commissioners require training, resources and shared learning opportunities to build their capacity to lead a holistic, whole‐systems approach. Any reform of the commissioning system must emphasise social value and earmark increased funding for prevention and public health approaches which are co‐designed, co‐delivered and co‐evaluated with, and by, service users and communities.

## AUTHOR CONTRIBUTIONS

**Rebecca J. Scott**: Methodology; writing—original draft; formal analysis. **Elspeth Mathie**: Conceptualisation; writing—review and editing. **Hannah J. H. Newman**: Conceptualization; writing—review and editing. **Kathryn Almack**: Writing—review and editing. **Louca‐Mai Brady**: Conceptualisation; funding acquisition; writing—review and editing.

## CONFLICT OF INTEREST STATEMENT

The authors declare no conflict of interest.

## Supporting information

Supporting information.

Supporting information.

## Data Availability

Data sharing is not applicable to this article as no new data were created or analyzed in this study. No datasets were generated or analysed for this study.
