# Supplementary material for: Commissioning and co‐production in health and care services in the United Kingdom and Ireland: An exploratory literature review
Source: Health Expect. 2024 May 2;27(3):e14053. doi: 10.1111/hex.14053 (PMC11066417; doi:10.1111/hex.14053)
Supplement: Supplementary file 1 — Supporting information. [file HEX-27-e14053-s001.docx]

Co-production and commissioning review: Final search strategies

# Ovid MEDLINE(R) ALL <1946 to March 15, 2023>

## Search run 16/03/2023

1 Community Participation/ 18381

2 Patient Participation/ 29217

3 1 or 2 47170

4 ((Patient* or public* or lay or people or consumer* or user* or citizen* or communit*) adj4 (participat* or involve* or engag*)).ti,ab. 183073

5 (health* or "social care" or "integrated care").ti,ab. 3460385

6 4 and 5 71190

7 ((health* or "social care") adj3 (partners or partnership)).ti,ab. 5211

8 3 or 6 or 7 115210

9 (co-design* or co-produc* or co-creat* or codesign* or coproduc* or cocreat*).tw. 11627

10 8 or 9 125310

11 commission*.ti,ab,tw. 48711

12 co-commission*.tw. 13

13 CCGs.ti,ab. 450

14 ((policymakers or policy makers) adj6 (contract* or fund* or purchas* or procure*)).ti,ab. 1062

title((policymakers or "policy makers") PRE/6 (contract* or fund* purchas* or procure*)) OR abstract((policymakers or "policy makers") PRE/6 (contract* or fund* purchas* or procure*))

15 ((NHS adj2 reform*) or (NHS adj2 transform*)).ti,ab. 494

16 "purchaser provider".ti,ab. 167

17 ((fund or funds or funded or funder or funders or funding) adj6 service*).ti,ab. 6341

18 ((fund or funds or funded or funder or funders or funding) adj2 contract*).ti,ab. 102

19 ((fund or funds or funded or funder or funders or funding) adj2 (provid* or provision*)).ti,ab. 2801

20 Decision Making, Organizational/ 11238

21 Health Priorities/og {no related terms} 485

22 Health Care Reform/mt, og {no related terms} 444

23 or/11-22 70909

24 (barrier* or challeng* or difficult* or obstacle* or driver* or enabler* or facilitat* or factor* or change* or issue* or constraint* or "in conflict" or conflicting or tension*).ti,ab. 9611948

25 10 and 23 and 24 1404

26 25 and 2008:2023.(sa_year). 949

27 ("royal commission" or "european commission" or "joint commission" or "trade commission" or "EU commission" or "judicial commission" or "federal commission" or "UN commission" or "global commission").ti,ab. 10221

28 ("regional commission" or "international commission").ti. 445

29 27 or 28 10666

30 26 not 29 906

31 afghanistan/ or africa/ or africa, northern/ or africa, central/ or africa, eastern/ or "africa south of the sahara"/ or africa, southern/ or africa, western/ or albania/ or algeria/ or andorra/ or angola/ or "antigua and barbuda"/ or argentina/ or armenia/ or azerbaijan/ or bahamas/ or bahrain/ or bangladesh/ or barbados/ or belize/ or benin/ or bhutan/ or bolivia/ or borneo/ or "bosnia and herzegovina"/ or botswana/ or brazil/ or brunei/ or bulgaria/ or burkina faso/ or burundi/ or cabo verde/ or cambodia/ or cameroon/ or central african republic/ or chad/ or exp china/ or comoros/ or congo/ or cote d'ivoire/ or croatia/ or cuba/ or "democratic republic of the congo"/ or cyprus/ or djibouti/ or dominica/ or dominican republic/ or ecuador/ or egypt/ or el salvador/ or equatorial guinea/ or eritrea/ or eswatini/ or ethiopia/ or fiji/ or gabon/ or gambia/ or "georgia (republic)"/ or ghana/ or grenada/ or guatemala/ or guinea/ or guinea-bissau/ or guyana/ or haiti/ or honduras/ or independent state of samoa/ or exp india/ or indian ocean islands/ or indochina/ or indonesia/ or iran/ or iraq/ or jamaica/ or jordan/ or kazakhstan/ or kenya/ or kosovo/ or kuwait/ or kyrgyzstan/ or laos/ or lebanon/ or liechtenstein/ or lesotho/ or liberia/ or libya/ or madagascar/ or malaysia/ or malawi/ or mali/ or malta/ or mauritania/ or mauritius/ or mekong valley/ or melanesia/ or micronesia/ or monaco/ or mongolia/ or montenegro/ or morocco/ or mozambique/ or myanmar/ or namibia/ or nepal/ or nicaragua/ or niger/ or nigeria/ or oman/ or pakistan/ or palau/ or exp panama/ or papua new guinea/ or paraguay/ or peru/ or philippines/ or qatar/ or "republic of belarus"/ or "republic of north macedonia"/ or romania/ or exp russia/ or rwanda/ or "saint kitts and nevis"/ or saint lucia/ or "saint vincent and the grenadines"/ or "sao tome and principe"/ or saudi arabia/ or serbia/ or sierra leone/ or senegal/ or seychelles/ or singapore/ or somalia/ or south africa/ or south sudan/ or sri lanka/ or sudan/ or suriname/ or syria/ or taiwan/ or tajikistan/ or tanzania/ or thailand/ or timor-leste/ or togo/ or tonga/ or "trinidad and tobago"/ or tunisia/ or turkmenistan/ or uganda/ or ukraine/ or united arab emirates/ or uruguay/ or uzbekistan/ or vanuatu/ or venezuela/ or vietnam/ or west indies/ or yemen/ or zambia/ or zimbabwe/ 1276665

32 "Organisation for Economic Co-Operation and Development"/ 515

33 australasia/ or exp australia/ or austria/ or baltic states/ or belgium/ or exp canada/ or chile/ or colombia/ or costa rica/ or czech republic/ or exp denmark/ or estonia/ or europe/ or finland/ or exp france/ or exp germany/ or greece/ or hungary/ or iceland/ or ireland/ or israel/ or exp italy/ or exp japan/ or korea/ or latvia/ or lithuania/ or luxembourg/ or mexico/ or netherlands/ or new zealand/ or north america/ or exp norway/ or poland/ or portugal/ or exp "republic of korea"/ or "scandinavian and nordic countries"/ or slovakia/ or slovenia/ or spain/ or sweden/ or switzerland/ or turkey/ or exp united kingdom/ or exp united states/ 3471417

34 European Union/ 17565

35 Developed Countries/ 21311

36 or/32-35 3487230

37 31 not 36 1187644

38 30 not 37 834

# Social Policy and Practice <202210> via Ovid

## Search run 16/03/2023

1 community participation.hw. 96

2 Patient Participation.hw. 5

3 ((Patient* or public* or lay or people or consumer* or user* or citizen* or communit*) adj4 (participat* or involve* or engag*)).ti,ab. 17515

4 (health* or "social care" or "integrated care").ti,ab. 122059

5 3 and 4 6551

6 1 or 2 101

7 ((health* or "social care") adj3 (partners or partnership)).ti,ab. 954

8 5 or 6 or 7 7513

9 (co-design* or co-produc* or co-creat* or codesign* or coproduc* or cocreat*).tw. 1826

10 commission*.tw. 21110

11 co-commission*.tw. 21

12 CCGs.ti,ab. 314

13 ((policymakers or policy makers) adj6 (contract* or fund* or purchas* or procure*)).ti,ab. 136

14 ((NHS adj2 reform*) or (NHS adj2 transform*)).ti,ab. 241

15 "purchaser provider".ti,ab. 169

16 ((fund or funds or funded or funder or funders or funding) adj6 service*).ti,ab. 3373

17 ((fund or funds or funded or funder or funders or funding) adj2 contract*).ti,ab. 67

18 ((fund or funds or funded or funder or funders or funding) adj2 (provid* or provision*)).ti,ab. 1082

19 ("organisational decision making" or "organizational decision making").ti,ab. 16

20 ("health care reform" or "healthcare reform").ti,ab. 71

21 "Health Priorities".ti,ab. 79

22 "care priorities".ti,ab. 29

23 8 or 9 9036

24 10 or 11 or 12 or 13 or 14 or 15 or 16 or 17 or 18 or 19 or 20 or 21 or 22 25374

25 (barrier* or challeng* or difficult* or obstacle* or driver* or enabler* or facilitat* or factor* or change* or issue* or constraint* or "in conflict" or conflicting or tension*).ti,ab. 190117

26 23 and 24 and 25 837

27 26 and 2008:2023.(sa_year). 678

28 ("royal commission" or "european commission" or "joint commission" or "trade commission" or "EU commission" or "judicial commission" or "federal commission" or "UN commission" or "global commission").ti,ab. 930

29 ("regional commission" or "international commission").ti. 0

30 28 or 29 930

31 27 not 30 676

# Public Health database via Proquest

## Search run 17/03/2023

**Search Strategy**

| Set# | Searched for | Databases | Results |
| --- | --- | --- | --- |
| S1 | mainsubject.Exact("community participation") | Public Health Database | 2396 |
| S2 | mainsubject.Exact("patient participation") | Public Health Database | 1476 |
| S3 | title((Patient* or public* or lay or people or consumer* or user* or citizen*) PRE/4 (participat* or involve* or engag* or communit*)) OR abstract((Patient* or public* or lay or people or consumer* or user* or citizen* or communit*) PRE/4 (participat* or involve* or engag*)) | Public Health Database | 30979 |
| S4 | title((health* or "social care" or "integrated care")) OR abstract((health* or "social care" or "integrated care")) | Public Health Database | 1489007 |
| S5 | title(((health* or "social care") PRE/3 (partners or partnership))) OR abstract(((health* or "social care") PRE/3 (partners or partnership))) | Public Health Database | 3815 |
| S6 | title((co-design* or co-produc* or co-creat* or codesign* or coproduc* or cocreat*)) OR abstract((co-design* or co-produc* or co-creat* or codesign* or coproduc* or cocreat*)) | Public Health Database | 2389 |
| S7 | S3 AND S4 | Public Health Database  These databases are searched for part of your query. | 16897 |
| S8 | S1 OR S2 OR S5 OR S6 OR S7 | Public Health Database  These databases are searched for part of your query. | 24612 |
| S9 | title(commission*) OR abstract(commission*) | Public Health Database | 56470 |
| S10 | title(co-commission*) OR abstract(co-commission*) | Public Health Database | 17 |
| S11 | title(CCGs) OR abstract(CCGs) | Public Health Database | 115 |
| S12 | title(((policymakers or "policy makers") PRE/6 (contract* or fund* or purchas* or procure*))) OR abstract(((policymakers or "policy makers") PRE/6 (contract* or fund* or purchas* or procure*))) | Public Health Database | 268 |
| S13 | title((NHS PRE/2 reform*) or (NHS PRE/2 transform*)) OR abstract((NHS PRE/2 reform*) or (NHS PRE/2 transform*)) | Public Health Database | 882 |
| S14 | title("purchaser provider") OR abstract("purchaser provider") | Public Health Database | 46 |
| S15 | title(((fund or funds or funded or funder or funders or funding) PRE/6 service*)) OR abstract(((fund or funds or funded or funder or funders or funding) PRE/6 service*)) | Public Health Database | 2638 |
| S16 | title(((fund OR funds OR funded OR funder OR funders OR funding) PRE/2 contract*)) OR abstract(((fund OR funds OR funded OR funder OR funders OR funding) PRE/2 contract*)) | Public Health Database | 99 |
| S17 | title(((fund OR funds OR funded OR funder OR funders OR funding) PRE/2 (provid* or provision*))) OR abstract(((fund OR funds OR funded OR funder OR funders OR funding) PRE/2 (provid* or provision*))) | Public Health Database | 1210 |
| S18 | mainsubject.Exact("decision making, organizational") | Public Health Database | 1174 |
| S19 | mainsubject.Exact("health priorities") | Public Health Database | 1195 |
| S20 | mainsubject.Exact("health care reform") | Public Health Database | 3611 |
| S21 | S9 OR S10 OR S11 OR S12 OR S13 OR S14 OR S15 OR S16 OR S17 OR S18 OR S19 OR S20 | Public Health Database  These databases are searched for part of your query. | 66961 |
| S22 | title(barrier* or challeng* or difficult* or obstacle* or driver* or enabler* or facilitat* or factor* or change* or issue* or constraint* or "in conflict" or conflicting or tension*) OR abstract(barrier* or challeng* or difficult* or obstacle* or driver* or enabler* or facilitat* or factor* or change* or issue* or constraint* or "in conflict" or conflicting or tension*) | Public Health Database | 1300921 |
| S23 | S8 AND S21 AND S22 | Public Health Database  These databases are searched for part of your query. | 378 |
| S24 | title(("royal commission" or "european commission" or "joint commission" or "trade commission" or "EU commission" or "judicial commission" or "federal commission" or "UN commission" or "global commission")) OR abstract(("royal commission" or "european commission" or "joint commission" or "trade commission" or "EU commission" or "judicial commission" or "federal commission" or "UN commission" or "global commission")) | Public Health Database | 8588 |
| S25 | title(("regional commission" or "international commission")) | Public Health Database | 81 |
| S26 | S24 OR S25 | Public Health Database  These databases are searched for part of your query. | 8669 |
| S27 | (S8 AND S21 AND S22) NOT (S24 OR S25) | Public Health Database  These databases are searched for part of your query. | 347 |
| S28 | ((S8 AND S21 AND S22) NOT (S24 OR S25)) AND pd(20080101-20230317) | Public Health Database  These databases are searched for part of your query. | 273 |
| S29 | ((S8 AND S21 AND S22) NOT (S24 OR S25)) AND (la.exact("ENG") AND pd(20080101-20230317)) | Public Health Database  These databases are searched for part of your query. | 272 |
| S30 | (S8 AND S21 AND S22) NOT (S24 OR S25) NOT (location.exact("Africa" OR "China" OR "India" OR "Tanzania" OR "Torres Strait" OR "Alexandria Egypt" OR "Angola" OR "Argentina" OR "Brazil" OR "Cape Verde Islands" OR "Central Asia" OR "Egypt" OR "Ghana" OR "Guatemala" OR "Indonesia" OR "Kenya" OR "Kyrgyzstan" OR "Lebanon" OR "Malawi" OR "Malta" OR "Middle East" OR "Nigeria" OR "North Africa" OR "Saudi Arabia" OR "South Africa" OR "Thailand" OR "Uganda" OR "Ukraine" OR "Union of Soviet Socialist Republics--USSR" OR "Zambia") AND la.exact("ENG") AND pd(20080101-20230317)) | Public Health Database  These databases are searched for part of your query. | 245 |

# Web of Science

(Arts & Humanities Citation Index, Social Sciences Citation Index, Science Citation Index Expanded, Conference Proceedings Citation Index – Science, Conference Proceedings Citation Index - Social Sciences & Humanities, Book Citation Index-Science, Book Citation Index-Social Sciences & Humanities, Emerging Sources Citation Index)

## Search run 16/03/2023

# Searches:

1: TS=(((Patient* or public* or lay or people or consumer* or user* or citizen* or communit*) NEAR/4 (participat* or involve* or engag*))) Editions: WOS.SCI,WOS.SSCI,WOS.AHCI,WOS.ISTP,WOS.ISSHP,WOS.BSCI,WOS.BHCI,WOS.ESCI Date Run: Thu Mar 16 2023 14:35:22 GMT+0000 (Greenwich Mean Time) Results: 333748

2: (TI=((health* or "social care" or "integrated care"))) OR AB=((health* or "social care" or "integrated care")) Editions: WOS.SCI,WOS.SSCI,WOS.AHCI,WOS.ISTP,WOS.ISSHP,WOS.BSCI,WOS.BHCI,WOS.ESCI Date Run: Thu Mar 16 2023 14:35:52 GMT+0000 (Greenwich Mean Time) Results: 3876102

3: #2 AND #1 Editions: WOS.SCI,WOS.SSCI,WOS.AHCI,WOS.ISTP,WOS.ISSHP,WOS.BSCI,WOS.BHCI,WOS.ESCI Date Run: Thu Mar 16 2023 14:36:10 GMT+0000 (Greenwich Mean Time) Results: 88192

4: (TI=(((health* or "social care") NEAR/3 (partners or partnership)))) OR AB=(((health* or "social care") NEAR/3 (partners or partnership))) Editions: WOS.SCI,WOS.SSCI,WOS.AHCI,WOS.ISTP,WOS.ISSHP,WOS.BSCI,WOS.BHCI,WOS.ESCI Date Run: Thu Mar 16 2023 14:36:32 GMT+0000 (Greenwich Mean Time) Results: 11706

5: TS=((co-design* or co-produc* or co-creat* or codesign* or coproduc* or cocreat*)) Editions: WOS.SCI,WOS.SSCI,WOS.AHCI,WOS.ISTP,WOS.ISSHP,WOS.BSCI,WOS.BHCI,WOS.ESCI Date Run: Thu Mar 16 2023 14:36:50 GMT+0000 (Greenwich Mean Time) Results: 57287

6: #3 OR #4 OR #5 Editions: WOS.SCI,WOS.SSCI,WOS.AHCI,WOS.ISTP,WOS.ISSHP,WOS.BSCI,WOS.BHCI,WOS.ESCI Date Run: Thu Mar 16 2023 14:37:10 GMT+0000 (Greenwich Mean Time) Results: 153768

7: TS=(commission*) Editions: WOS.SCI,WOS.SSCI,WOS.AHCI,WOS.ISTP,WOS.ISSHP,WOS.BSCI,WOS.BHCI,WOS.ESCI Date Run: Thu Mar 16 2023 14:37:23 GMT+0000 (Greenwich Mean Time) Results: 147199

8: TS=(co-commission*) Editions: WOS.SCI,WOS.SSCI,WOS.AHCI,WOS.ISTP,WOS.ISSHP,WOS.BSCI,WOS.BHCI,WOS.ESCI Date Run: Thu Mar 16 2023 14:37:39 GMT+0000 (Greenwich Mean Time) Results: 25

9: (TI=(CCGs)) OR AB=(CCGs) Editions: WOS.SCI,WOS.SSCI,WOS.AHCI,WOS.ISTP,WOS.ISSHP,WOS.BSCI,WOS.BHCI,WOS.ESCI Date Run: Thu Mar 16 2023 14:38:00 GMT+0000 (Greenwich Mean Time) Results: 578

10: (TI=(NHS NEAR/2 reform*)) OR AB=(NHS NEAR/2 reform*) Editions: WOS.SCI,WOS.SSCI,WOS.AHCI,WOS.ISTP,WOS.ISSHP,WOS.BSCI,WOS.BHCI,WOS.ESCI Date Run: Thu Mar 16 2023 14:38:15 GMT+0000 (Greenwich Mean Time) Results: 470

11: (TI=(NHS NEAR/2 transform*)) OR AB=(NHS NEAR/2 transform*) Editions: WOS.SCI,WOS.SSCI,WOS.AHCI,WOS.ISTP,WOS.ISSHP,WOS.BSCI,WOS.BHCI,WOS.ESCI Date Run: Thu Mar 16 2023 14:38:33 GMT+0000 (Greenwich Mean Time) Results: 53

12: (TI=("purchaser provider")) OR AB=("purchaser provider") Editions: WOS.SCI,WOS.SSCI,WOS.AHCI,WOS.ISTP,WOS.ISSHP,WOS.BSCI,WOS.BHCI,WOS.ESCI Date Run: Thu Mar 16 2023 14:38:52 GMT+0000 (Greenwich Mean Time) Results: 180

13: AK=("organisational decision making" OR "organizational decision making") Editions: WOS.SCI,WOS.SSCI,WOS.AHCI,WOS.ISTP,WOS.ISSHP,WOS.BSCI,WOS.BHCI,WOS.ESCI Date Run: Thu Mar 16 2023 14:39:10 GMT+0000 (Greenwich Mean Time) Results: 144

14: AK=("healthcare reform" ) Editions: WOS.SCI,WOS.SSCI,WOS.AHCI,WOS.ISTP,WOS.ISSHP,WOS.BSCI,WOS.BHCI,WOS.ESCI Date Run: Thu Mar 16 2023 14:39:25 GMT+0000 (Greenwich Mean Time) Results: 496

15: (TI=((policymakers or "policy makers") NEAR/6 (contract* or fund* or purchas* or procure*))) OR AB=((policymakers or "policy makers") NEAR/6 (contract* or fund* or purchas* or procure*)) Editions: WOS.SCI,WOS.SSCI,WOS.AHCI,WOS.ISTP,WOS.ISSHP,WOS.BSCI,WOS.BHCI,WOS.ESCI Date Run: Thu Mar 16 2023 14:40:19 GMT+0000 (Greenwich Mean Time) Results: 2066

16: (TI=(((fund or funds or funded or funder or funders or funding) NEAR/6 service*))) OR AB=(((fund or funds or funded or funder or funders or funding) NEAR/6 service*)) Editions: WOS.SCI,WOS.SSCI,WOS.AHCI,WOS.ISTP,WOS.ISSHP,WOS.BSCI,WOS.BHCI,WOS.ESCI Date Run: Thu Mar 16 2023 14:41:30 GMT+0000 (Greenwich Mean Time) Results: 9056

17: (TI=(((fund or funds or funded or funder or funders or funding) NEAR/2 contract*))) OR AB=(((fund or funds or funded or funder or funders or funding) NEAR/2 contract*)) Editions: WOS.SCI,WOS.SSCI,WOS.AHCI,WOS.ISTP,WOS.ISSHP,WOS.BSCI,WOS.BHCI,WOS.ESCI Date Run: Thu Mar 16 2023 14:42:09 GMT+0000 (Greenwich Mean Time) Results: 536

18: (TI=(((fund or funds or funded or funder or funders or funding) NEAR/2 (provid* or provision*)))) OR AB=(((fund or funds or funded or funder or funders or funding) NEAR/2 (provid* or provision*))) Editions: WOS.SCI,WOS.SSCI,WOS.AHCI,WOS.ISTP,WOS.ISSHP,WOS.BSCI,WOS.BHCI,WOS.ESCI Date Run: Thu Mar 16 2023 14:43:13 GMT+0000 (Greenwich Mean Time) Results: 6920

19: #18 OR #17 OR #16 OR #15 OR #14 OR #13 OR #12 OR #11 OR #10 OR #9 OR #8 OR #7 Editions: WOS.SCI,WOS.SSCI,WOS.AHCI,WOS.ISTP,WOS.ISSHP,WOS.BSCI,WOS.BHCI,WOS.ESCI Date Run: Thu Mar 16 2023 14:43:52 GMT+0000 (Greenwich Mean Time) Results: 165822

20: (TI=((barrier* or challeng* or difficult* or obstacle* or driver* or enabler* or facilitat* or factor* or change* or issue* or constraint* or "in conflict" or conflicting or tension*))) OR AB=((barrier* or challeng* or difficult* or obstacle* or driver* or enabler* or facilitat* or factor* or change* or issue* or constraint* or "in conflict" or conflicting or tension*)) Editions: WOS.SCI,WOS.SSCI,WOS.AHCI,WOS.ISTP,WOS.ISSHP,WOS.BSCI,WOS.BHCI,WOS.ESCI Date Run: Thu Mar 16 2023 14:45:11 GMT+0000 (Greenwich Mean Time) Results: 17948236

21: #20 AND #19 AND #6 Editions: WOS.SCI,WOS.SSCI,WOS.AHCI,WOS.ISTP,WOS.ISSHP,WOS.BSCI,WOS.BHCI,WOS.ESCI Date Run: Thu Mar 16 2023 14:45:50 GMT+0000 (Greenwich Mean Time) Results: 1438

22: ALL=("royal commission" or "european commission" or "joint commission" or "trade commission" or "EU commission" or "judicial commission" or "federal commission" or "UN commission" or "global commission") Editions: WOS.SCI,WOS.SSCI,WOS.AHCI,WOS.ISTP,WOS.ISSHP,WOS.BSCI,WOS.BHCI,WOS.ESCI Date Run: Thu Mar 16 2023 14:46:36 GMT+0000 (Greenwich Mean Time) Results: 797146

23: TI=( "regional commission" or "international commission") Editions: WOS.SCI,WOS.SSCI,WOS.AHCI,WOS.ISTP,WOS.ISSHP,WOS.BSCI,WOS.BHCI,WOS.ESCI Date Run: Thu Mar 16 2023 14:47:01 GMT+0000 (Greenwich Mean Time) Results: 715

24: #22 OR #23 Editions: WOS.SCI,WOS.SSCI,WOS.AHCI,WOS.ISTP,WOS.ISSHP,WOS.BSCI,WOS.BHCI,WOS.ESCI Date Run: Thu Mar 16 2023 14:47:11 GMT+0000 (Greenwich Mean Time) Results: 797860

25: #21 NOT #24 Editions: WOS.SCI,WOS.SSCI,WOS.AHCI,WOS.ISTP,WOS.ISSHP,WOS.BSCI,WOS.BHCI,WOS.ESCI Date Run: Thu Mar 16 2023 14:47:39 GMT+0000 (Greenwich Mean Time) Results: 1260

26: #25 Editions: WOS.SCI,WOS.SSCI,WOS.AHCI,WOS.ISTP,WOS.ISSHP,WOS.BSCI,WOS.BHCI,WOS.ESCI Timespan: 2008-01-01 to 2023-03-16 Date Run: Thu Mar 16 2023 14:48:51 GMT+0000 (Greenwich Mean Time) Results: 1108

27: #25 and ZIMBABWE or VENEZUELA or UKRAINE or TUNISIA or SUDAN or SINT MAARTEN or SERBIA or RWANDA or NIGER or NAMIBIA or MAURITIUS or KYRGYZSTAN or KUWAIT or GAMBIA or EGYPT or BULGARIA or BERMUDA or BELARUS or BAHRAIN or ZAMBIA or VIETNAM or UZBEKISTAN or SRI LANKA or SENEGAL or REP CONGO or TANZANIA or BURKINA FASO or MALAWI or MOZAMBIQUE or QATAR or CAMBODIA or CAMEROON or ETHIOPIA or LEBANON or U ARAB EMIRATES or ARGENTINA or DEM REP CONGO or KAZAKHSTAN or SAUDI ARABIA or GHANA or UGANDA or PEOPLES R CHINA or SOUTH AFRICA or INDIA or BRAZIL or KENYA or NIGERIA or RUSSIA or IRAN or TAIWAN or INDONESIA or PAKISTAN or SINGAPORE or CROATIA or MALAYSIA or ROMANIA or BANGLADESH or ECUADOR or IRAQ or NEPAL or PERU or PHILIPPINES or THAILAND (Exclude – Countries/Regions) Editions: WOS.SCI,WOS.SSCI,WOS.AHCI,WOS.ISTP,WOS.ISSHP,WOS.BSCI,WOS.BHCI,WOS.ESCI Timespan: 2008-01-01 to 2023-03-16 Date Run: Thu Mar 16 2023 15:03:18 GMT+0000 (Greenwich Mean Time) Results: 932
