# Supplementary material for: Commissioning and co‐production in health and care services in the United Kingdom and Ireland: An exploratory literature review
Source: Health Expect. 2024 May 2;27(3):e14053. doi: 10.1111/hex.14053 (PMC11066417; doi:10.1111/hex.14053)
Supplement: Supplementary file 2 — Supporting information. [file HEX-27-e14053-s002.docx]

# Appendix 1: Included studies

Table 2: List of included studies

| **Document type** | **Authors** | **Year** | **Country** | **Approach** | **Method** | **Context** |
| --- | --- | --- | --- | --- | --- | --- |
| Journal Article | Alderson H, Kaner E, O’Donnell A, Bate A.^55^ | 2022 | England | Service user involvement | Qualitative | Addiction services |
| Journal Article | Patterson S, Weaver T, Agath K, et al.^56^ | 2009 | England | Service user involvement | Qualitative | Addiction services |
| Journal Article | Crone D, Ellis L, Bryan H, Pearce M, Ford J.^57^ | 2021 | England | Co-production | Qualitative | Arts for health |
| Journal Article | Attree P, Morris S, Payne S, Vaughan S, Hinder S.^87^ | 2011 | UK | Service user involvement | Qualitative | Cancer care |
| Journal Article | Coultas C, Kieslich K, Littlejohns P.^58^ | 2019 | England | Patient and Public Involvement | Qualitative | Commissioning |
| Journal Article | O'Shea A, Boaz AL, Chambers M.^59^ | 2019 | England | Patient and Public Involvement | Qualitative | Commissioning |
| Journal Article | Hatfield D, Aranda K, Ferns G, Flaherty B, Hart A.^60^ | 2023 | England | Patient and Public Involvement | Qualitative | Commissioning |
| Report | Baird B, Cream J, Weaks L.^89^ | 2018 | UK | Co-production | Qualitative | Commissioning |
| Report | University Of Bristol Norah Fry Centre for Disability Studies.^88^ | 2018 | UK | Co-production | Qualitative | Commissioning |
| Journal Article | Hart F.^90^ | 2022 | UK | Co-production | Opinion piece | Commissioning |
| Report | Schehrer S, Sexton S.^61^ | 2010 | England | Service user involvement | Qualitative | Commissioning |
| Report | Calovski V, Taylor F, O'Shea A, Brearley S, Chambers M.^62^ | 2020 | England | Patient and Public Involvement | Qualitative | Commissioning |
| Journal Article | Evans DH, Bacon RJ, Greer E, Stagg AM, Turton P.^63^ | 2013 | England | Service user involvement | Qualitative | Commissioning |
| Report | Boelman V, Russell C.^64^ | 2013 | England | Co-production | Qualitative | Disabilities |
| Report | Bernd S.^65^ | 2012 | England | User-driven commissioning | Qualitative | Disabilities |
| Report | Moss A, Miller R, Battye F, et al.^66^ | 2018 | England | Co-production | Qualitative | Disabilities |
| Report | Hampson M, Baeck P, Langford K.^67^ | 2013 | England | Co-design and co-delivery | Qualitative | Healthcare systems |
| Journal Article | Peckham S, Wilson P, Williams L, et al.^68^ | 2014 | England | Patient and Public Involvement | Qualitative | Long-term conditions |
| Report | Ballantyne P, Temperley J.^69^ | 2016 | England | Co-production | Qualitative | Mental health |
| Journal Article | Baxter L, Fancourt D.^91^ | 2020 | UK | Lived experience | Qualitative | Mental health |
| Journal Article | Davie E.^70^ | 2012 | England | Service user involvement | Literature review | Mental health |
| Report | Kalathil J.^71^ | 2013 | England | Service user involvement | Qualitative | Mental health |
| Report | Durcan G, Stubbs J, Appleton S, Bell A.^72^ | 2017 | England | Co-production | Qualitative | Mental health |
| Report | Minghella E, Linsky K.^73^ | 2018 | England | Co-production | Qualitative | Mental health |
| Journal Article | McEvoy P, Williamson T, Kada R, Frazer D, Dhliwayo C, Gask L.^74^ | 2017 | England | Community engagement | Mixed methods | Mental health |
| Journal Article | McKeown M, Jones F, Wright K, et al.^75^ | 2016 | England | Service user involvement | Qualitative | Mental health |
| Report | National Development Team For Inclusion.^76^ | 2019 | England | Co-production | Qualitative | Mental health |
| Report | Sainsbury Centre For Mental Health.^77^ | 2010 | England | Service user involvement | Qualitative | Mental health |
| Report | Thiel V, Sonola L, Goodwin N, Kodner DL.^78^ | 2013 | England | Co-production | Qualitative | Mental health |
| Report | Ageing Better^92^ | 2018 | UK | Community connector model | Mixed methods | Older adults |
| Report | Wigfield A, Alden S.^79^ | 2017 | England | Co-production | Mixed methods | Older adults |
| Theses | Whitehead L.^80^ | 2020 | England | Co-production | Qualitative | Older adults |
| Journal Article | Chadborn N, Craig C, Sands G, Schneider J, Gladman J.^81^ | 2019 | England | Co-production | Qualitative | Older adults |
| Report | Close L.^97^ | 2017 | Scotland | Co-production | Qualitative | Older adults |
| Journal Article | Davies A, French DP, Devereux-Fitzgerald A, et al.^82^ | 2021 | England | Participatory approaches | Qualitative | Older adults |
| Journal Article | McEvoy R, MacFarlane A.^99^ | 2013 | Ireland | Community participation | Qualitative | Primary care |
| Report | Pillinger J.^100^ | 2010 | Ireland | Community participation | Mixed methods | Primary care |
| Journal Article | Snooks HA, Evans BA, Cohen D, et al.^101^ | 2011 | Wales | Participatory approaches | Qualitative | Public health |
| Report | Boyle D, Slay J, Stephens L.^93^ | 2010 | UK | Co-production | Qualitative | Public services |
| Report | Boyle D, Coote A, Sherwood C, Slay J.^94^ | 2010 | UK | Co-production | Qualitative | Public services |
| Journal Article | Loeffler E, Bovaird T. C^95^ | 2019 | UK | Co-production | Model | Public services |
| Journal Article | Early F, Winders S-J, Reddy S, Ralphs J, Fuld J.^83^ | 2017 | England | Co-production | Qualitative | Respiratory diseases |
| Journal Article | Farmer J, Currie M, Kenny A, Munoz S-A.^98^ | 2015 | Scotland | Community participation | Qualitative | Rural health |
| Report | Bedford S, Harper A.^84^ | 2018 | England | Community-led business | Qualitative | Social care |
| Report | Co-Operative College, Change Agents.^96^ | 2017 | UK | Co-operative approaches | Qualitative | Social care |
| Journal Article | Udayaraj UP, Watson O, Ben-Shlomo Y, et al.^85^ | 2019 | England | Co-design | Qualitative | Transplant |
| Journal Article | Osborne AK, McGill G, Greaves PJ, Kiernan MD.^86^ | 2022 | England | Co-design | Qualitative | Veteran services |
